# Supplementary material for: Cytogenetic screening of chromosomal abnormalities and genetic analysis of FSH receptor Ala307Thr and Ser680Asn genes in amenorrheic patients
Source: PeerJ. 2023 May 26;11:e15267. doi: 10.7717/peerj.15267 (PMC10226477; doi:10.7717/peerj.15267)
Supplement: Supplemental Information 1 [file peerj-11-15267-s001.pdf]

CYTOGENETIC SCREENING OF CHROMOSOMAL ABNORMALITIES AND GENETIC ANALYSIS OF FSH RECEPTOR ALA307THR AND SER680ASN GENES IN AMENORRHEIC PATIENTS

Questionnaires :-

- **Case number :-** ☐ **Date :** ☐
- **Name :-** ☐ **Age :-** ☐
- **Phone number :-** ☐
- **Weight :-** ☐ **Height :-** ☐ **BMI :-** ☐
- **WBCs count :-** ☐

- **Type of amenorrhea :-**

☐ **Primary amenorrhea** ☐

☐ **Secondary amenorrhea** ☐

  - **Absence menstrual cycle for : Three months :** ☐ **Six months :** ☐

- **Secondary sexual characteristics :** ☐
- **A family history of the age of menarche and menopause of the patient's mother and siblings or PCOs**

  ____

- **Ultrasonography**
  - **Ovaries :-**____
  - **Uterus :-** ____
  - **Endometrium:-** ____
  - **Cervix :-** ____
  - **Vagina :-** ____
- **MRI :-** ____
- **Anosmia :-** ☐
- **Hormonal assay :-**

FSH ☐ **LH** ☐ **E2** ☐ **PRL** ☐ **TSH** ☐

Testosterone ☐ **AMH** ☐
