# Supplementary material for: Cytogenetic screening of chromosomal abnormalities and genetic analysis of FSH receptor Ala307Thr and Ser680Asn genes in amenorrheic patients
Source: PeerJ. 2023 May 26;11:e15267. doi: 10.7717/peerj.15267 (PMC10226477; doi:10.7717/peerj.15267)
Supplement: Supplemental Information 4 [file peerj-11-15267-s004.pdf]

An official website of the United States government

Here's how you know

Log in

Nucleotide

GenBank

Homo sapiens 6_Ala307Thr FSHR gene for follicle stimulating hormone receptor, partial sequence

GenBank: LC739720.1

FASTA Graphics

Go to:

```
LOCUS       LC739720                 507 bp    DNA     linear   PRI 22-NOV-2022
DEFINITION  Homo sapiens 6_Ala307Thr FSHR gene for follicle stimulating hormone
            receptor, partial sequence.
ACCESSION   LC739720
VERSION     LC739720.1
KEYWORDS    .
SOURCE      Homo sapiens (human)
  ORGANISM  Homo sapiens
            Eukaryota; Metazoa; Chordata; Craniata; Vertebrata; Euteleostomi;
            Mammalia; Eutheria; Euarchontoglires; Primates; Haplorrhini;
            Catarrhini; Hominidae; Homo.
REFERENCE   1
  AUTHORS   Al-Ouqaili,M.T. and Kanaan,B.A.
  TITLE     Cytogenetic screening of chromosomal abnormalities and genetic
            analysis of FSH receptor Ala307Thr and Ser680Asn genes in
            amenorrheic patients
  JOURNAL   Unpublished
REFERENCE   2  (bases 1 to 507)
  AUTHORS   Al-Ouqaili,M.T. and Kanaan,B.A.
  TITLE     Direct Submission
  JOURNAL   Submitted (18-NOV-2022) Contact:Mushtak T. Al-Ouqaili College of
            Medicine- University of Al-Anbar, Department of Microbiology;
            Al-Anbar, Al-Anbar 31001, Iraq
FEATURES             Location/Qualifiers
     source          1..507
                     /organism="Homo sapiens"
                     /mol_type="genomic DNA"
                     /isolate="6_Ala307Thr"
                     /db_xref="taxon:9606"
                     /country="Iraq"
                     /collection_date="2022-09-15"
                     /collected_by="Mushtak T.S.Al-Ouqaili and Bushra A.
                     kanaan"
                     /note="MBA-Ala"
     gene            <1..>507
                     /gene="FSHR"
     misc_feature    <1..>507
                     /gene="FSHR"
                     /note="follicle stimulating hormone receptor"
ORIGIN
        1 tgatgtattt gctatactgg atctgagatg ttgattctat ttctttttgt atttttctag
       61 ctctgagctt catccaattt gcaacaaatc tattttaagg caagaagttg attatatgac
      121 tcagrctagg ggtcagagat cctctctggc agaagacaat gagtccagct acagcagagg
      181 atttgacatg acgtacactg agtttgacta tgacttatgc aatgaagtgg ttgacgtgac
      241 ctgctcccct aagccagatg cattcaaccc atgtgaagat atcatggggt acaacatcct
      301 cagagtcctg atatggttta tcagcatcct ggccatcact gggaacatca tagtgctagt
      361 gatcctaact accagccaat ataaactcac agtccccagg ttccttatgt gcaacctggc
      421 ctttgctgat ctctgcattg gaatctacct gctgctcatt gcatcagttg atatccatac
      481 caagagccaa tatcacaact atgccat
//
```
