# Supplementary material for: Cytogenetic screening of chromosomal abnormalities and genetic analysis of FSH receptor Ala307Thr and Ser680Asn genes in amenorrheic patients
Source: PeerJ. 2023 May 26;11:e15267. doi: 10.7717/peerj.15267 (PMC10226477; doi:10.7717/peerj.15267)
Supplement: Supplemental Information 8 [file peerj-11-15267-s008.pdf]

An official website of the United States government
Here's how you know

Log in

Nucleotide

GenBank

Homo sapiens 6_Ser680Asn FSHR gene for follicle stimulating hormone receptor, partial sequence

GenBank: LC739724.1

FASTA Graphics

Go to:

```
LOCUS       LC739724                 448 bp    DNA     linear   PRI 22-NOV-2022
DEFINITION  Homo sapiens 6_Ser680Asn FSHR gene for follicle stimulating hormone
            receptor, partial sequence.
ACCESSION   LC739724
VERSION     LC739724.1
KEYWORDS    .
SOURCE      Homo sapiens (human)
  ORGANISM  Homo sapiens
            Eukaryota; Metazoa; Chordata; Craniata; Vertebrata; Euteleostomi;
            Mammalia; Eutheria; Euarchontoglires; Primates; Haplorrhini;
            Catarrhini; Hominidae; Homo.
REFERENCE   1
  AUTHORS   Al-Ouqaili,M.T. and Kanaan,B.A.
  TITLE     Cytogenetic screening of chromosomal abnormalities and genetic
            analysis of FSH receptor Ala307Thr and Ser680Asn genes in
            amenorrheic patients
  JOURNAL   Unpublished
REFERENCE   2  (bases 1 to 448)
  AUTHORS   Al-Ouqaili,M.T. and Kanaan,B.A.
  TITLE     Direct Submission
  JOURNAL   Submitted (18-NOV-2022) Contact:Mushtak T. Al-Ouqaili College of
            Medicine- University of Al-Anbar, Department of Microbiology;
            Al-Anbar, Al-Anbar 31001, Iraq
FEATURES             Location/Qualifiers
     source          1..448
                     /organism="Homo sapiens"
                     /mol_type="genomic DNA"
                     /isolate="6_Ser680Asn"
                     /db_xref="taxon:9606"
                     /country="Iraq"
                     /collection_date="2022-09-15"
                     /collected_by="Mushtak T.S.Al-Ouqaili and Bushra A.
                     kanaan"
                     /note="MBA-Ser"
     gene            <1..>448
                     /gene="FSHR"
     misc_feature    <1..>448
                     /gene="FSHR"
                     /note="follicle stimulating hormone receptor"
ORIGIN
        1 cgtgtcctcc tctagtgaca ccaggatcgc caagcgcatg gccatgctca tcttcactga
       61 cttcctctgc atggcaccca tttctttctt tgccatttct gcctccctca aggtgcccct
      121 catcactgtg tccaaagcaa agattctgct ggttctgttt caccccatca actcctgtgc
      181 caaccccttc ctctatgcca tctttaccaa aaactttcgc agagatttct tcattctgct
      241 gagcaagtgt ggctgctatg aaatgcaagc ccaaatttat aggacagaaa cttcatccac
      301 tgtccacaac acccatccaa ggaatggcca ctgctcttca gctcccagag tcaccaatgg
      361 ttccacttac atacttgtcc ctctaagtca tttagcccaa aactaaaaca caatgtgaaa
      421 atgtatctga gtattgaatg ataattca
//
```
