# Supplementary material for: Cytogenetic screening of chromosomal abnormalities and genetic analysis of FSH receptor Ala307Thr and Ser680Asn genes in amenorrheic patients
Source: PeerJ. 2023 May 26;11:e15267. doi: 10.7717/peerj.15267 (PMC10226477; doi:10.7717/peerj.15267)
Supplement: Supplemental Information 10 [file peerj-11-15267-s010.docx]

|  | Name | Type of amenorrhea | Age | BMI | FSH | LH |
| --- | --- | --- | --- | --- | --- | --- |
| 1 | P1 | PA | 20 | 24.1 | 0.1 | 0.2 |
| 2 | P2 | PA | 16 | 31.5 | 58.3 | 14.7 |
| 3 | P3 | PA | 17 | 25.7 | 5.8 | 4.6 |
| 4 | P4 | PA | 16 | 27.6 | 0.5 | 0.1 |
| 5 | P5 | PA | 14 | 16.0 | 9.7 | 3.55 |
| 6 | P6 | PA | 15 | 27.4 | 105.0 | 54.89 |
| 7 | P7 | PA | 19 | 30.1 | 63.2 | 10.6 |
| 8 | P8 | PA | 21 | 30.5 | 1.3 | 1.1 |
| 9 | P9 | PA | 25 | 26.3 | 59.9 | 6.78 |
| 10 | P10 | PA | 17 | 21.6 | 6.1 | 4.7 |
| 11 | P11 | PA | 16 | 19.3 | 73.25 | 35.6 |
| 12 | P12 | PA | 14 | 24.8 | 11.6 | 3.5 |
| 13 | P13 | PA | 21 | 27.3 | 52.2 | 9.5 |
| 14 | P14 | PA | 15 | 20.7 | 8.1 | 5.9 |
| 15 | P15 | PA | 16 | 26.0 | 56.9 | 17.4 |
| 16 | P16 | PA | 18 | 22.8 | 67.3 | 7.4 |
| 17 | P17 | PA | 16 | 28.0 | 45.7 | 10.31 |
| 18 | P18 | PA | 15 | 29.1 | 1.54 | 0.69 |
| 19 | P19 | PA | 17 | 30.5 | 98.1 | 44.6 |
| 20 | P20 | PA | 21 | 20.7 | 65.2 | 15.9 |
| 21 | P21 | PA | 20 | 31.0 | 19.6 | 4.7 |
| 22 | P22 | PA | 23 | 25.9 | 107.8 | 34.29 |
| 23 | P23 | PA | 25 | 19.1 | 14.7 | 5.22 |
| 24 | P24 | PA | 14 | 28.9 | 6.3 | 2.1 |
| 25 | P25 | PA | 19 | 21.6 | 78.4 | 38.1 |
| 26 | P26 | PA | 15 | 20.3 | 53.2 | 6.65 |
| 27 | P27 | PA | 18 | 22.5 | 45.9 | 13.2 |
| 28 | P28 | PA | 17 | 24.6 | 52.7 | 11.23 |
| 29 | P29 | PA | 16 | 16.8 | 0.7 | 3.1 |
| 30 | P30 | PA | 19 | 29.0 | 8.34 | 5.43 |
| 31 | P31 | PA | 22 | 21.5 | 121.76 | 29.7 |
| 32 | P32 | PA | 14 | 20.6 | 98.3 | 24.32 |
| 33 | P33 | PA | 16 | 20.3 | 59.98 | 8.98 |
| 34 | P34 | PA | 18 | 16.9 | 88.7 | 15.4 |
| 35 | P35 | PA | 21 | 26.0 | 68.5 | 4.37 |
| 36 | P36 | PA | 16 | 19.4 | 121.2 | 27.59 |
| 37 | P37 | PA | 17 | 27.3 | 45.9 | 34.41 |
| 38 | P38 | PA | 19 | 18.3 | 65.8 | 13.56 |
| 39 | P39 | PA | 15 | 21.7 | 6.1 | 3.35 |
| 40 | P40 | PA | 20 | 25.2 | 53.9 | 25.4 |

Data of Table 2

| n | Name | Type of amenorrhea | Age | BMI | FSH | LH |
| --- | --- | --- | --- | --- | --- | --- |
|  | P1 | SA | 27 | 21.7 | 2.3 | 4.5 |
|  | P2 | SA | 36 | 27.5 | 65.6 | 8.39 |
|  | P3 | SA | 30 | 31.4 | 11.1 | 13.0 |
|  | P4 | SA | 22 | 33.4 | 8.7 | 1.2 |
|  | P5 | SA | 20 | 27.3 | 52.37 | 7.61 |
|  | P6 | SA | 18 | 20.6 | 141.0 | 6.8 |
|  | P7 | SA | 26 | 23.9 | 114.4 | 45.0 |
|  | P8 | SA | 23 | 27.9 | 3.1 | 0.5 |
|  | P9 | SA | 28 | 21.8 | 96.46 | 18.0 |
|  | P10 | SA | 21 | 34.0 | 35.5 | 3.4 |
|  | P11 | SA | 21 | 25.0 | 6.8 | 11.7 |
|  | P12 | SA | 35 | 28.5 | 53.1 | 20.1 |
|  | P13 | SA | 24 | 23.8 | 32.9 | 9.4 |
|  | P14 | SA | 21 | 31.7 | 52.5 | 3.8 |
|  | P15 | SA | 27 | 20.9 | 12.1 | 11.8 |
|  | P16 | SA | 33 | 24.7 | 7.0 | 7.1 |
|  | P17 | SA | 29 | 32.0 | 6.2 | 4.9 |
|  | P18 | SA | 26 | 20.8 | 98.4 | 15.9 |
|  | P19 | SA | 23 | 29.4 | 10.3 | 11.6 |
|  | P20 | SA | 25 | 28.1 | 38.7 | 5.1 |
|  | P21 | SA | 34 | 22.7 | 45.34 | 12.1 |
|  | P22 | SA | 30 | 24.9 | 27.9 | 26.4 |
|  | P23 | SA | 22 | 19.5 | 120.0 | 47.6 |
|  | P24 | SA | 32 | 21.3 | 9.4 | 9.8 |
|  | P25 | SA | 24 | 26.6 | 18.3 | 10.8 |
|  | P26 | SA | 20 | 26.4 | 67.1 | 16.5 |
|  | P27 | SA | 35 | 30.4 | 35.5 | 3.7 |
|  | P28 | SA | 23 | 32.1 | 110.2 | 35.6 |
|  | P29 | SA | 27 | 26.5 | 15.2 | 5.8 |
|  | P30 | SA | 26 | 22.8 | 18.5 | 7.5 |

| N | Number of Healthy Control Women | AGE | BMI | FSH | LH | Karyotype Results |
| --- | --- | --- | --- | --- | --- | --- |
|  | C1 | 31 | 26.0 | 6.02 | 5.2 | Normal |
|  | C2 | 27 | 19.3 | 6.9 | 4.1 | Normal |
|  | C3 | 15 | 20.7 | 5.9 | 7.5 | Normal |
|  | C4 | 33 | 21.5 | 5.8 | 9.02 | Normal |
|  | C5 | 28 | 25.4 | 5.06 | 3.4 | Normal |
|  | C6 | 16 | 20.3 | 5.0 | 3.6 | Normal |
|  | C7 | 24 | 26.7 | 4.8 | 7.6 | Normal |
|  | C8 | 30 | 22.1 | 4.6 | 2.4 | Normal |
|  | C9 | 22 | 23.9 | 5.05 | 5.2 | Normal |
|  | C10 | 20 | 19.8 | 5.06 | 5.4 | Normal |
|  | C11 | 25 | 20.0 | 5.0 | 3.7 | Normal |
|  | C12 | 22 | 25.2 | 5.4 | 3.8 | Normal |
|  | C13 | 18 | 28.5 | 6.6 | 4.7 | Normal |
|  | C14 | 21 | 23.4 | 7.2 | 7.1 | Normal |
|  | C15 | 19 | 24.0 | 6.6 | 4.9 | Normal |
|  | C16 | 23 | 20.2 | 7.0 | 7.1 | Normal |
|  | C17 | 21 | 22.0 | 6.2 | 4.9 | Normal |
|  | C18 | 24 | 27.4 | 8.3 | 4.7 | Normal |
|  | C19 | 27 | 25.2 | 5.1 | 9.4 | Normal |
|  | C20 | 17 | 22.8 | 7.2 | 4.8 | Normal |
|  | C21 | 26 | 29.4 | 6.3 | 4.1 | Normal |
|  | C22 | 19 | 23.5 | 5.5 | 3.9 | Normal |
|  | C23 | 16 | 20.7 | 7.0 | 7.5 | Normal |
|  | C24 | 30 | 28.0 | 5.1 | 2.8 | Normal |
|  | C25 | 25 | 25.4 | 4.9 | 5.3 | Normal |
|  | C26 | 14 | 21.0 | 6.1 | 7.0 | Normal |
|  | C27 | 21 | 24.2 | 5.7 | 4.4 | Normal |
|  | C28 | 24 | 25.1 | 5.2 | 3.9 | Normal |
|  | C29 | 17 | 20.3 | 4.7 | 2.5 | Normal |
|  | C30 | 20 | 22.8 | 7.1 | 6.8 | Normal |
