# Supplementary material for: Cytogenetic screening of chromosomal abnormalities and genetic analysis of FSH receptor Ala307Thr and Ser680Asn genes in amenorrheic patients
Source: PeerJ. 2023 May 26;11:e15267. doi: 10.7717/peerj.15267 (PMC10226477; doi:10.7717/peerj.15267)
Supplement: Supplemental Information 13 [file peerj-11-15267-s013.docx]

|  | Patients Number | Type of Amenorrhea | Age | BMI | FSH  (20<) | LH | Karyotype Results | Genotypes of Ser680Asn (rs6166) |
| --- | --- | --- | --- | --- | --- | --- | --- | --- |
| 1 | P6 | PA | 15 | 27.4 | 105.0 | 54.89 | Normal | AG |
| 2 | P7 | PA | 19 | 30.1 | 63.2 | 10.6 | Normal | AG |
| 3 | P9 | PA | 25 | 26.3 | 59.9 | 6.78 | Normal | GG |
| 4 | P11 | PA | 16 | 19.3 | 73.25 | 35.6 | Normal | AG |
| 5 | P13 | PA | 21 | 27.3 | 52.2 | 9.5 | Normal | AA |
| 6 | P15 | PA | 16 | 26.0 | 56.9 | 17.4 | Normal | AG |
| 7 | P16 | PA | 18 | 22.8 | 67.3 | 7.4 | Normal | GG |
| 8 | P17 | PA | 16 | 28.0 | 45.7 | 10.31 | Normal | AG |
| 9 | P22 | PA | 23 | 25.9 | 107.8 | 34.29 | Normal | GG |
| 10 | P27 | PA | 18 | 22.5 | 45.9 | 13.2 | Normal | AG |
| 11 | P28 | PA | 17 | 24.6 | 52.7 | 11.23 | Normal | AA |
| 12 | P31 | PA | 22 | 21.5 | 121.76 | 29.7 | Normal | AG |
| 13 | P32 | PA | 14 | 20.6 | 98.3 | 24.32 | Normal | AA |
| 14 | P33 | PA | 16 | 20.3 | 59.98 | 8.98 | Normal | AG |
| 15 | P34 | PA | 18 | 16.9 | 88.7 | 15.4 | Normal | AG |
| 16 | P35 | PA | 21 | 26.0 | 68.5 | 4.37 | Normal | AA |
| 17 | P37 | PA | 17 | 27.3 | 45.9 | 34.41 | Normal | GG |
| 18 | P40 | PA | 20 | 25.2 | 53.9 | 25.4 | Normal | AG |

Data of Table 9

| N | Number of Healthy Control Women | AGE | BMI | FSH | LH | Karyotype Results | Genotype of Ser680Asn (rs6166) |
| --- | --- | --- | --- | --- | --- | --- | --- |
|  | C1 | 31 | 26.0 | 6.02 | 5.2 | Normal | GG |
|  | C2 | 27 | 19.3 | 6.9 | 4.1 | Normal | AA |
|  | C3 | 15 | 20.7 | 5.9 | 7.5 | Normal | AA |
|  | C4 | 33 | 21.5 | 5.8 | 9.02 | Normal | AA |
|  | C5 | 28 | 25.4 | 5.06 | 3.4 | Normal | AG |
|  | C6 | 16 | 20.3 | 5.0 | 3.6 | Normal | AA |
|  | C7 | 24 | 26.7 | 4.8 | 7.6 | Normal | AG |
|  | C8 | 30 | 22.1 | 4.6 | 2.4 | Normal | AG |
|  | C9 | 22 | 23.9 | 5.05 | 5.2 | Normal | AG |
|  | C10 | 20 | 19.8 | 5.06 | 5.4 | Normal | GG |
|  | C11 | 25 | 20.0 | 5.0 | 3.7 | Normal | AA |
|  | C12 | 22 | 25.2 | 5.4 | 3.8 | Normal | AA |
|  | C13 | 18 | 28.5 | 6.6 | 4.7 | Normal | AA |
|  | C14 | 21 | 23.4 | 7.2 | 7.1 | Normal | AA |
|  | C15 | 19 | 24.0 | 6.6 | 4.9 | Normal | AA |
|  | C16 | 23 | 20.2 | 7.0 | 7.1 | Normal | AG |
|  | C17 | 21 | 22.0 | 6.2 | 4.9 | Normal | AA |
|  | C18 | 24 | 27.4 | 8.3 | 4.7 | Normal | AG |
|  | C19 | 27 | 25.2 | 5.1 | 9.4 | Normal | AG |
|  | C20 | 17 | 22.8 | 7.2 | 4.8 | Normal | AG |
|  | C21 | 26 | 29.4 | 6.3 | 4.1 | Normal | GG |
|  | C22 | 19 | 23.5 | 5.5 | 3.9 | Normal | AA |
|  | C23 | 16 | 20.7 | 7.0 | 7.5 | Normal | AG |
|  | C24 | 30 | 28.0 | 5.1 | 2.8 | Normal | AG |
|  | C25 | 25 | 25.4 | 4.9 | 5.3 | Normal | AA |
|  | C26 | 14 | 21.0 | 6.1 | 7.0 | Normal | AA |
|  | C27 | 21 | 24.2 | 5.7 | 4.4 | Normal | AA |
|  | C28 | 24 | 25.1 | 5.2 | 3.9 | Normal | AG |
|  | C29 | 17 | 20.3 | 4.7 | 2.5 | Normal | AA |
|  | C30 | 20 | 22.8 | 7.1 | 6.8 | Normal | AA |
