# Supplementary material for: Cytogenetic screening of chromosomal abnormalities and genetic analysis of FSH receptor Ala307Thr and Ser680Asn genes in amenorrheic patients
Source: PeerJ. 2023 May 26;11:e15267. doi: 10.7717/peerj.15267 (PMC10226477; doi:10.7717/peerj.15267)
Supplement: Supplemental Information 15 [file peerj-11-15267-s015.docx]

|  | Patients Number | Type of Amenorrhea | Age | BMI | FSH  (20<) | LH | Karyotype Results | Genotypes of Ala307Thr (rs6165) | Genotypes of Ser680Asn (rs6166) |
| --- | --- | --- | --- | --- | --- | --- | --- | --- | --- |
| 1 | P6 | PA | 15 | 27.4 | 105.0 | 54.89 | Normal | AA | AG |
| 2 | P7 | PA | 19 | 30.1 | 63.2 | 10.6 | Normal | AG | AG |
| 3 | P9 | PA | 25 | 26.3 | 59.9 | 6.78 | Normal | AA | GG |
| 4 | P11 | PA | 16 | 19.3 | 73.25 | 35.6 | Normal | AA | AG |
| 5 | P13 | PA | 21 | 27.3 | 52.2 | 9.5 | Normal | AA | AA |
| 6 | P15 | PA | 16 | 26.0 | 56.9 | 17.4 | Normal | AA | AG |
| 7 | P16 | PA | 18 | 22.8 | 67.3 | 7.4 | Normal | AG | GG |
| 8 | P17 | PA | 16 | 28.0 | 45.7 | 10.31 | Normal | AG | AG |
| 9 | P22 | PA | 23 | 25.9 | 107.8 | 34.29 | Normal | AG | GG |
| 10 | P27 | PA | 18 | 22.5 | 45.9 | 13.2 | Normal | GG | AG |
| 11 | P28 | PA | 17 | 24.6 | 52.7 | 11.23 | Normal | AA | AA |
| 12 | P31 | PA | 22 | 21.5 | 121.76 | 29.7 | Normal | AA | AG |
| 13 | P32 | PA | 14 | 20.6 | 98.3 | 24.32 | Normal | AA | AA |
| 14 | P33 | PA | 16 | 20.3 | 59.98 | 8.98 | Normal | AG | AG |
| 15 | P34 | PA | 18 | 16.9 | 88.7 | 15.4 | Normal | GG | AG |
| 16 | P35 | PA | 21 | 26.0 | 68.5 | 4.37 | Normal | AG | AA |
| 17 | P37 | PA | 17 | 27.3 | 45.9 | 34.41 | Normal | AG | GG |
| 18 | P40 | PA | 20 | 25.2 | 53.9 | 25.4 | Normal | AA | AG |

**Table: Cytogenetic variation, karyotype and frequency of patients with primary amenorrhea.**

**Case:** A 17-years-old girl had a PA with delay puberal. Physical examination, short statue, short and webbed neck, hands and feet that were swollen and wide chest. On the general examination, her tall is 150 cm, weight 68.6 Kg and her BMI was 30.5kg/m2. Familial history of menarche was normal.

Examination of secondary sexual characteristics which showed no breast development, auxiliary and pubic hair were absent. Ultrasound scanning results showed don’t have typical ovarian development which appeared streak ovaries.

 In an investigation of a hormonal assay, measured serum of FSH and LH were found to be very high, 98.1and 44.6 mIU /ml, respectively.

The chromosomal study analysis (Karyotype) was done which showed 45, X karyotype with monosomy X in all metaphases analyzed.


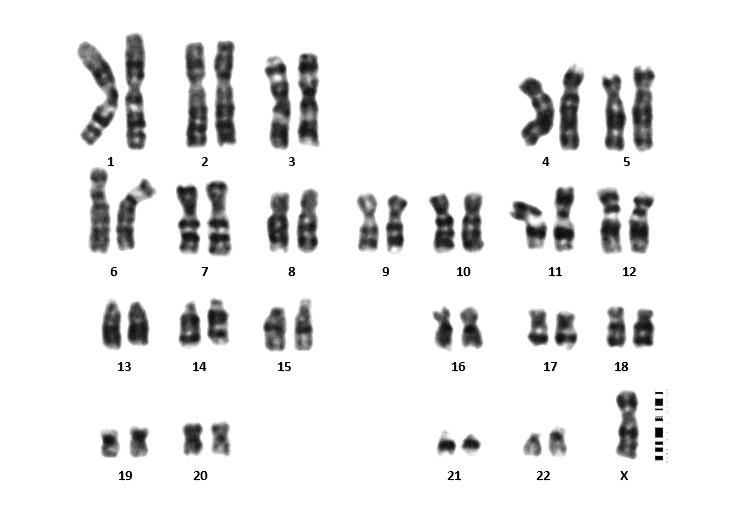


**Karyotype**: 45,X.

**Result:** Monosomy X in all metaphase analysis.

Case: A 16 years old girl with primary amenorrhea. There was normal family history, and all of them achieved their menarche at a convenient age. On general examination, tall was 147 cm, weight was 68 kg body mass index (BMI) was 31.5 kg/m2.

 Examination of secondary sexual characteristics, development of breast (Tanner stage I), and without axillary and pubic hair. The external genitalia of her were exanimated, which appears that as female type. Ultrasound showed, infantile uterus measuring 8.8cc uterus showed no focal lesion, endometrial thickness 2mm, and both ovaries was not detected.

 In an investigation of a hormonal assay, measured serum of FSH and LH were found to be very high,58 and 14.7 mIU /ml, respectively.

The chromosomal study analysis (Karyotype) was done which showed 46,Xi(X)(q10) karyotype in all metaphases analyzed which is consistent with variant of Turner syndrome.

**Karyotype:** 45,X /46,Xi(X)(q10).

**Result:** Mosaicism with two cell lines, the first line show monosomy X represent 60%, the second line show isochromosome X of long arm represent 40%.

**Case**: A 21-years-old female with a complaint of Primary amenorrhea. History of this case, without abdominal pain, radiation exposure, immunological disease, chemotherapy, surgical history, or any central nervous symptoms such as visual disorder and headache. There was normal family history, and all of them achieved their menarche at a convenient age. On general examination, tall was 157 cm, weight was 51 kg body mass index (BMI) was 20.7 kg/m2.

 Examination of secondary sexual characteristics, development of breast (Tanner stage Ⅲ), and axillary and pubic hair were sparse (Tanner stage I). The external genitalia of her were exanimated, which appears that as female type. The vaginal and cervix appeared normal. CT scan showed the size of the uterus was normal, and streak ovaries were evident.

 In an investigation of a hormonal assay, measured serum of FSH and LH were found to be very high,65.2 and 15.9 mIU /ml, respectively.

The chromosomal study analysis (Karyotype) was done which showed male karyotype in all metaphases analyzed 46, XY (Swyer syndrome).


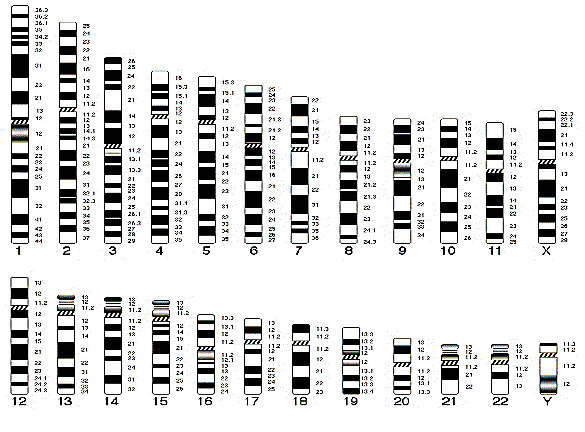

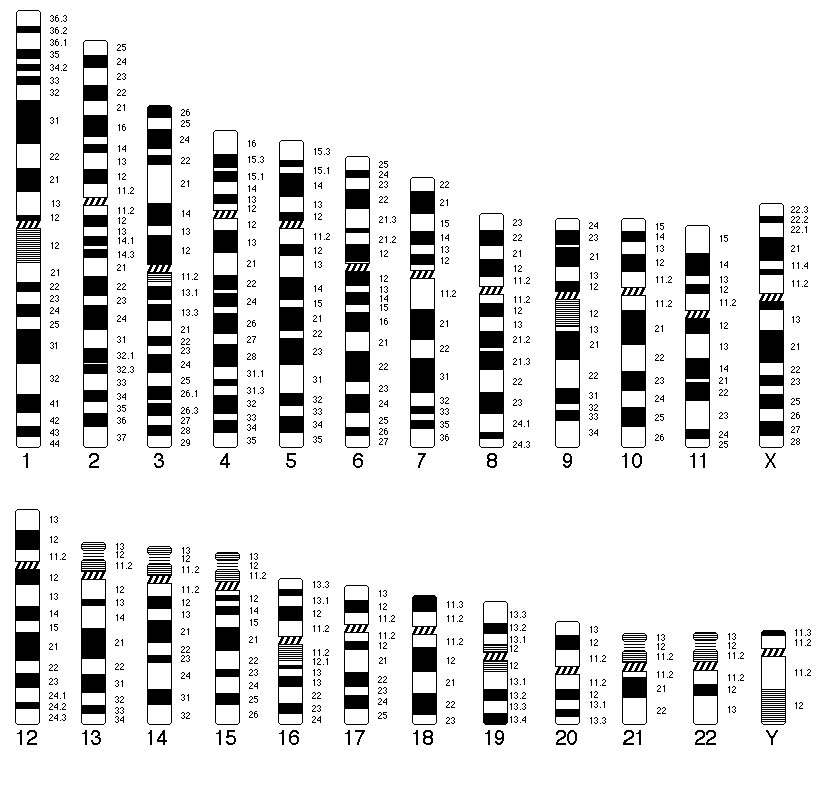

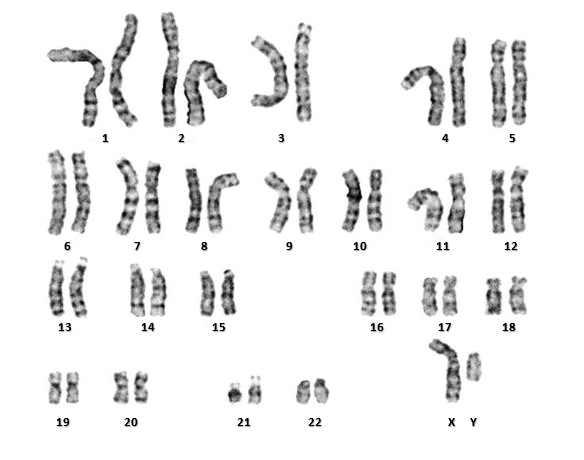


**Karyotype:** 46,XY

**Result:** Male karyotype in all metaphases analyzed.

Case: A 20-years-old female which suffering from Primary amenorrhea. A family history of the age of menarche and menopause of patient’s mother and siblings was normal. On general examination, tall was 168 cm, weight was 68 kg body mass index (BMI) was 24.1 kg/m2.

 Examination of secondary sexual characteristics, development of breast (Tanner stage Ⅲ), and axillary and pubic hair (Tanner stage II). Ultrasound showed the size of the uterus and uterus were small size while MRI was normal.

 In an investigation of a hormonal assay, measured serum of FSH and LH were found to be very low,0.1 and 0.2 mIU /ml, respectively.

The chromosomal study analysis (Karyotype) was done which showed 46,XX,-3,+der3,-19,del 19 p karyotype in all metaphases analyzed.

**Case 5**: A 19-year-old female with PA. On general examination, tall was 155 cm, weight was 52 kg body mass index (BMI) was 21.6 kg/m2.

Hormonal evaluation revealed hypergonadotropic hypogonadism, measured serum of FSH and LH were found to be very high,78.4 and 38.1 mIU /ml, respectively.

Physical examination revealed short stature, and pubic hair and breast development. Ultrasound revealed Streak ovaries and infantile uterus.

The chromosomal study analysis (Karyotype) was done which showed [45,X /46,Xi(X)(q10) ] karyotype in all metaphases analyzed. Mosaicism with two cell lines, the first line show monosomy X represent 60%, the second line show isochromosome X of long arm represent 40%. Diagnosis as Turner Syndrome Variant (Isochromosome Xq in Mosaic Turner Syndrome)


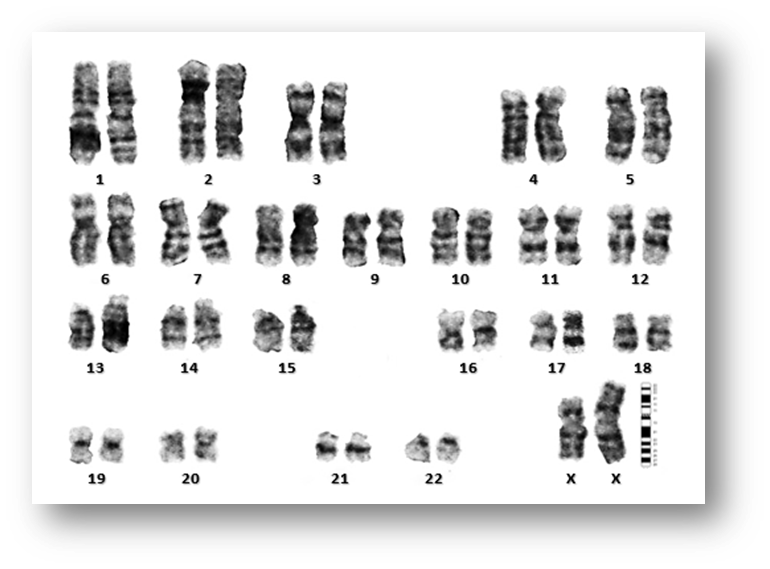


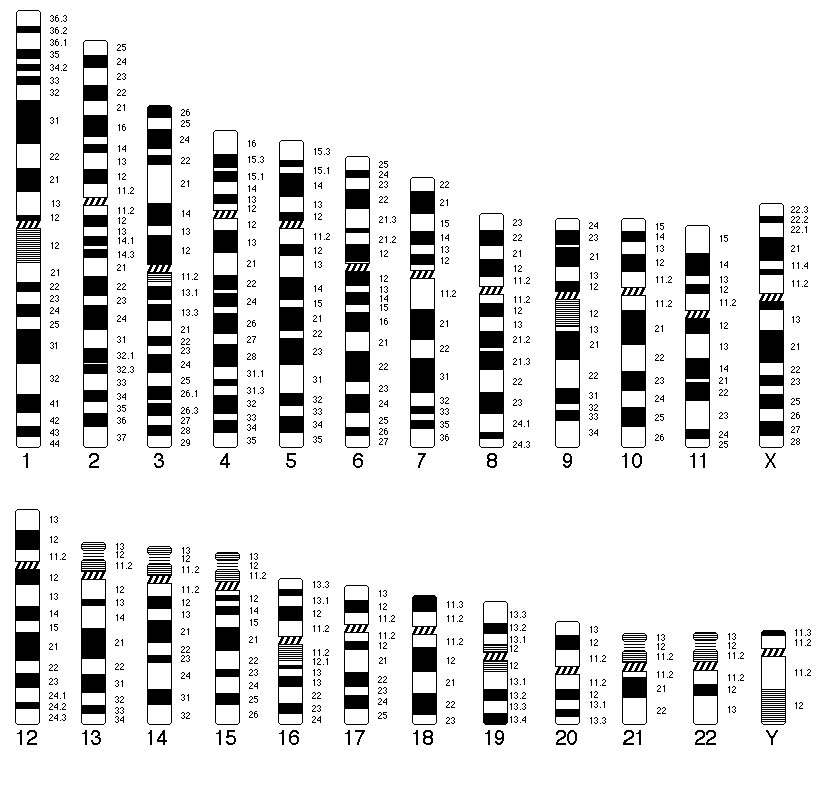


**19 20 21 22 X**

**13 14 15 16 17 18**

**6 7 8 9 10 11 12**

**1 2 3 4 5**
